# Supplementary material for: Predicted Effector Gene Aggregation, Standards and Unified Schema (PEGASUS): A Community Framework for Effector Gene Reporting
Source: bioRxiv. 2026 Jun 17:2026.06.16.731894. Preprint. [Version 1] doi: 10.64898/2026.06.16.731894 (PMC13307929; doi:10.64898/2026.06.16.731894)
Supplement: Supplement 2 — Supplementary Table 1: Evidence categories & definitions [file media-2.docx]

## Supplementary Table 1

| Supplementary Table 1a. Variant-centric evidence. | | |
| --- | --- | --- |
| Evidence categories | Abbreviation | Explanation |
| Linkage disequilibrium | LD | Assessment of whether a variant is correlated with another variant of interest and may act as a proxy. |
| Finemapping and credible sets | FM | Finemapping results - probability of variant being causal within a credible set, using Bayesian or probabilistic models |
| Colocalisation | COLOC | Variant affects two traits (typically a complex trait and a molecular phenotype) at the same locus. |
| Molecular QTL | QTL | Variant affects a molecular phenotype, e.g. gene expression (eQTL), splicing (sQTL), or protein expression (pQTL). |
| Mendelian Randomisation (MR) | MR | Uses genetic variants as proxies for exposures to test their causal effect on outcomes. |
| Regulatory region | REG | Variant lies in open chromatin or enhancer/promoter elements in relevant tissue (e.g. ATAC-seq, DNase-seq, or histone mark data) |
| Chromatin interaction | CHROMATIN | Variant lies in a region physically interacting with a gene promoter via 3D chromatin architecture (e.g. Hi-C, Capture-C data). |
| Predicted functional impact | FUNC | Variant predicted to disrupt gene/protein function or regulatory motifs, e.g. via SIFT, PolyPhen, CADD. |
| Proximity to gene (distance) | PROX | Assessment of whether variant is within or near gene boundaries. |
| Genome-wide association (GWAS) signal | GWAS | P-value from source GWAS for association of variant with trait specified in metadata file |
| PheWAS (Phenome-Wide Association Study) | PHEWAS | Variant is associated with multiple traits, suggesting pleiotropic effects |
| Cross-phenotype* | CROSSP | Gene or variant already established in a related phenotype (biologically similar). |
| Literature curation* | LIT | Human-curated gene or variant–disease links from literature. |
| Association from curated database* | DB | Variant or Gene is curated as causal or related to the phenotype from existing databases, like ClinVar, ClinGen, OMIM, etc. |

Evidence categories marked with * can also serve as gene-centric evidence

| Supplementary Table 1b. Gene-centric evidence | | |
| --- | --- | --- |
| Evidence categories | Abbreviation | Explanation |
| Protein–protein interaction | PPI | Gene’s protein interacts with other disease-relevant proteins. |
| Pathway or gene sets | SET | Gene is part of a known pathway or complex relevant to the phenotype, e.g. results of enrichment analyses using Reactome or KEGG. |
| Gene-based association | GENEBASE | Aggregated analysis of association of variants in gene with trait (e.g. SKAT, MAGMA, burden tests) . |
| Expression | EXP | Gene is differentially expressed in relevant tissue or disease e.g. the gene is more highly expressed in phenotype-related tissues compared to others. |
| Perturbation | PERTURB | Gene perturbation causes phenotype-relevant effects in lab or model organisms (knock out animal/cell line, human organoid). |
| Biological Knowledge Inference | KNOW | Gene–phenotype relationships can be inferred based on known biology, without providing specific references or direct experimental evidence linking the specific gene to the phenotype. |
| Genetically predicted trait association (TWAS/PWAS) | TPWAS | Evidence from transcriptome- or proteome-wide association studies showing that gene’s genetically predicted expression or protein level is associated with phenotype, |
| Drug related | DRUG | Evidence from drugs’ mechanism of action, e.g. gene encodes a known drug target or interacts with targets of drugs used to treat the phenotype, supporting therapeutic relevance. |
| Cross-phenotype* | CROSSP | Gene or variant already established in a related phenotype (biologically similar). |
| Literature curation* | LIT | Human-curated gene or variant–disease links from literature. |
| Association from curated database* | DB | Variant or Gene is curated as causal or related to the phenotype from existing databases, like ClinVar, ClinGen, OMIM, etc. |
|  |  |  |

Evidence categories marked with * can also serve as variant-centric evidence.
